# Recycled Steel Slag as a Porous Adsorbent to Filter Phosphorus-Rich Water with 8 Filtration Circles

**DOI:** 10.3390/ma14123187

**Published:** 2021-06-09

**Authors:** Han Lee, Yen-Ling Peng, Liang-Ming Whang, Jiunn-Der Liao

**Affiliations:** 1 Department of Materials Science and Engineering, National Cheng Kung University, 1 University Road, Tainan 701, Taiwan; 10608102@gs.ncku.edu.tw (H.L.); ye953g@gmail.com (Y.-L.P.); 2 Department of Environmental Engineering, National Cheng Kung University, 1 University Road, Tainan 701, Taiwan; whang@mail.ncku.edu.tw

**Keywords:** porous steel slag disc, phosphorus-rich water, phosphorus removal, filtration times, circular economy

## Abstract

Steel slag is a secondary product from steelmaking process through alkaline oxygen furnace or electric arc furnace (EAF). The disposal of steel slag has become a thorny environmental protection issue, and it is mainly used as unbound aggregates, e.g., as a secondary component of asphalt concrete used for road paving. In this study, the characteristics of compacted porous steel slag disc (SSD) and its application in phosphorous (P)-rich water filtration are discussed. The SSD with an optimal porosity of 10 wt% and annealing temperature of 900 °C, denoted as SSD-P (10, 900) meets a compressive strength required by ASTM C159-06, which has the capability of much higher than 90% P removal (with the effluent standard < 4 mg P/L) within 3 h, even after eight filtration times. No harmful substances from SSD have been detected in the filtered water, which complies with the effluent standard ISO 14001. The reaction mechanism for P-rich water filtration is mediated by water, followed by two reaction steps—CaO in SSD hydrolyzed from the matrix of SSD to Ca^2+^ and reacting with PO_4_^3−^. However, the microenvironment of water is influenced by the pH value of the P-rich water at different filtration times and the kind of P-rich water with different free positive ion that interferes the reactions of the release of Ca^2+^. This study demonstrates the application of circular economy in reducing steel slag deposits, filtering P-rich water, and collecting Ca_3_(PO_4_)_2_ precipitate into fertilizers.

## 1. Introduction

Circular economy aims to get rid of waste and use the resources in an efficient way [1,2]. This economic model utilizes reuse, remanufacturing and recycling to produce a closed-loop system, in order to reduce the use of resources to prevent pollution and carbon emissions. Therefore, waste recycling is an attractive disposal means to realize resource preservation, the costs and pollution issues can be minimized [3].

It has been reported that steel slag can be used for the removal of dyes and ions due to its effective adsorption effect [4,5]. As an unconventional adsorbent for various heavy metal ions, alkali slag combines ion exchange and adsorption characteristics and acid neutralization capabilities. The affinity of steel slag for phosphorus (P) ion binding has been studied to use by-products of the steel industry as a filter matrix to treat wastewater. Steel slag is obtained by the oxidation of steel pellets in an EAF [6]; it accounts for 15~20% of iron production. For example, the USA produces about 5.1 million tons of steel slag in a year [7]; in Europe every year nearly 12 million tons of steel slags are produced [8]. In fact, in some countries in the world, such as India [9] or Brazil [10], the utilization rate of this by-product is relatively low. Most of them are deposited in the slag storage field, thus causing much serious environmental trouble [6,11], e.g., soil and groundwater pollution. Steel slag is a by-product of the process of converting iron into steel, and it varies according to the raw material and process. To be precise, the EAF is a more modern method of steel production. Common inputs for converter steelmaking are iron ore and coal, while the EAF furnaces operates with scrap steel and have been produced and are ready for recycling [12,13]. Because the chemical composition of steel slags is changeable, it consists of CaO (45~60%), SiO_2_ (10~15%), Al_2_O_3_ (1~5%), Fe_2_O_3_ (3~9%), FeO (7~20%), and MgO (3~13%) [14]. Steel slag is widely used as aggregate in many countries. This does not completely consume the huge production volume and hence increasing amounts of steel slag are accumulating. Until today nearly 65% of the produced steel slags are used on a certified paddock of application in Europe. However, still, the existing 35% of these slags are landfilled [15]. Therefore, how to effectively solve this problem is extremely important. 

It is well-known that wastewater drainages are main sources of P-ion loads in waters such as rivers, lakes or lagoons [16]. The outcome usually leads to the enrichment of P in the receiving water body, which promotes the abnormal growth of algae and aquatic plants [17]. As aquatic plants and algae die, they decay by microbial decomposition decrease the concentration of dissolved oxygen. This phenomenon of water quality degradation as a result of excessive intake of P is commonly alluding to as eutrophication [18]. Therefore, laws on P released into the surrounding environment is becoming stricter throughout the world, including for wastewater treatment plants such as ISO 14001, European Union Directives 2000/60/EC, 91/271/EEC [19]. Chemical precipitation techniques are commonly used for P removal, owing to its cost–benefit. These methods exist for the removal of toxic metal ions from aqueous solution, as such ion exchange, reverse osmosis, precipitation, and adsorption, among others [20]. Adsorption is by far the most versatile and widely used process. Removing toxic metal-ions through adsorption is rather effective; however, its use is somewhat restricted due to the higher cost of activated carbon and difficulties associated with regeneration [21,22]. Attempts have therefore been made to utilize natural and waste materials as substitute adsorbents. However, the application of industrial waste materials is vital over the past few years because these wastes represent unused resources and, in a large number of cases, cause serious disposal problems. [23,24].

The purpose of using recycled materials containing steel slag as adsorbents is to provide multiple advantages in terms of environmental pollution [25,26]. First, the volume of waste can be partially reduced. Second, low-cost adsorbents (if developed) can reduce wastewater pollution at a reasonable cost. Considering the low cost of such adsorbents, there is no need to regenerate used materials [25,26]. Therefore, it is important to develop a low-cost adsorbent with a high surface area, which shows good adsorption potential to remove aqueous contaminants. In this regard, porous steel slag has potential. However, it is still a challenge to select the appropriate pore size and size for the porous structure and have satisfactory adsorption properties (e.g., pore connectivity, adsorption). Consequently, there is an urgent need to manufacture filters with physical strength and varying porosity [27].

In this study, a powder metallurgy (P/M) method is used for material processing to prepare porous steel slag absorbers [28], in which the evaluation of pore connectivity, pore size, and adsorption will be examined in detail [29]. Apart from this, P is a key non-renewable mineral essential for sustainable crop production. Therefore, a pragmatic method to efficiently recover P from wastewater is in high demand [30,31,32]. As an ecofriendly method, industrial wastewater is used as an adsorbent, which can be selectively separated from simulated wastewater through a permeation process to recover P from P-rich water [33,34,35].

## 2. Experimental 

### 2.1. Preparation of Porous Steel Slag Disc SSD_P (x, y)

**Figure 1:**
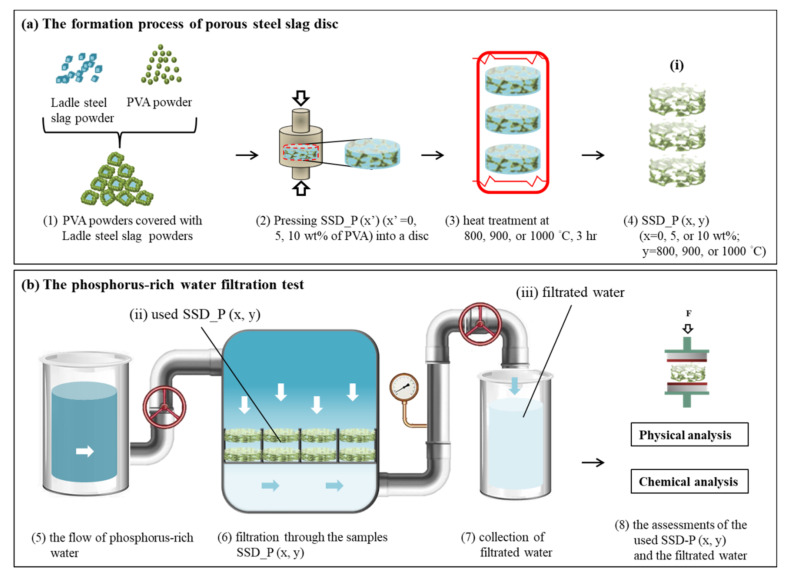
(**a**) The formation process of porous steel slag disc (SSD_P): (1) mixing Ladle steel slag powder with 0, 5, 10 wt% of PVA powder; (2) pressing SSD_P (x’) (x’ =0, 5, 10 wt% of PVA) into a disc; (3) SSD_P (x’) was followed by heat-treated at 800, 900, or 1000 °C for 3 h; (4) the as-heated samples were denoted as SSD_P (x, y) (x = 0, 5 or 10%, y = 800, 900 or 1000 °C). (**b**) The P-rich water filtration test: (5) the flow of P-rich water; (6) the filtration process through SSD_P (x, y); (7) the collection of filtrated water; (8) the examinations of the used SSD-P (x, y) and the filtrated water.

**Figure 2:**
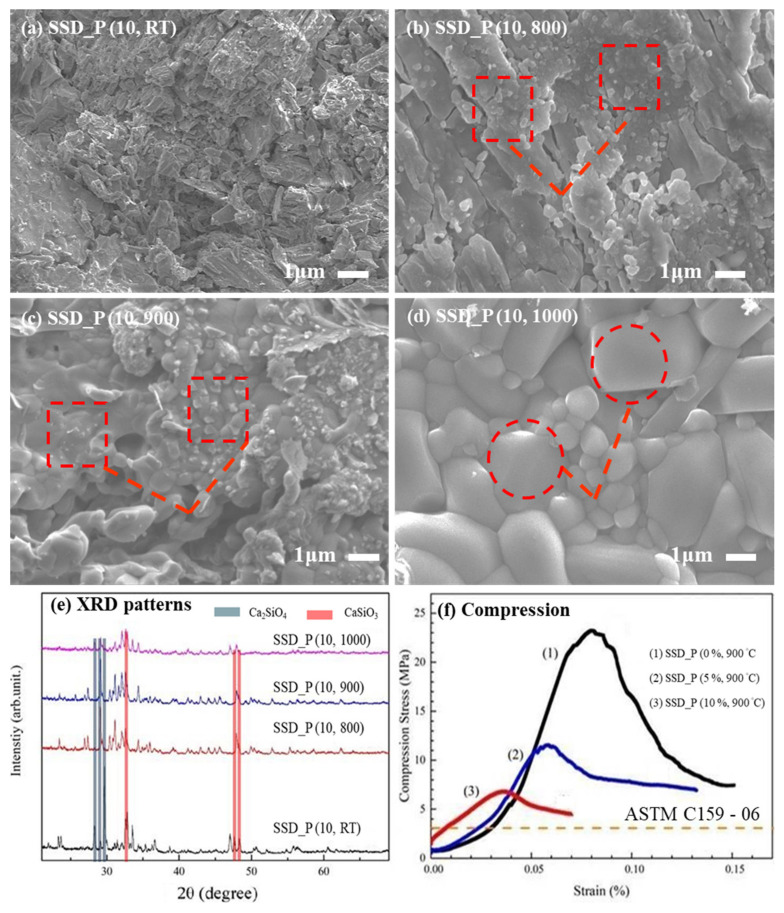
SEM morphologies from the surfaces of SSD_P (x, y), where x is 10% and y is annealed at (**a**) room temperature, (**b**) 800, (**c**) 900, and (**d**) 1000 °C, respectively. As the annealing temperature increases, recrystallization (e.g., marked in squares) and then grain growth (e.g., marked in circles) of SSD_P (x, y) can be observed. (**e**) XRD patterns and (**f**) the measured compression stress of SSD_P (10%, 900 °C) are measured by taking SSD_P (0%, 900 °C) and SSD_P (5%, 900 °C) as the references. A required strength based on ASTM C159-06 [38] is also shown.

**Figure 3:**
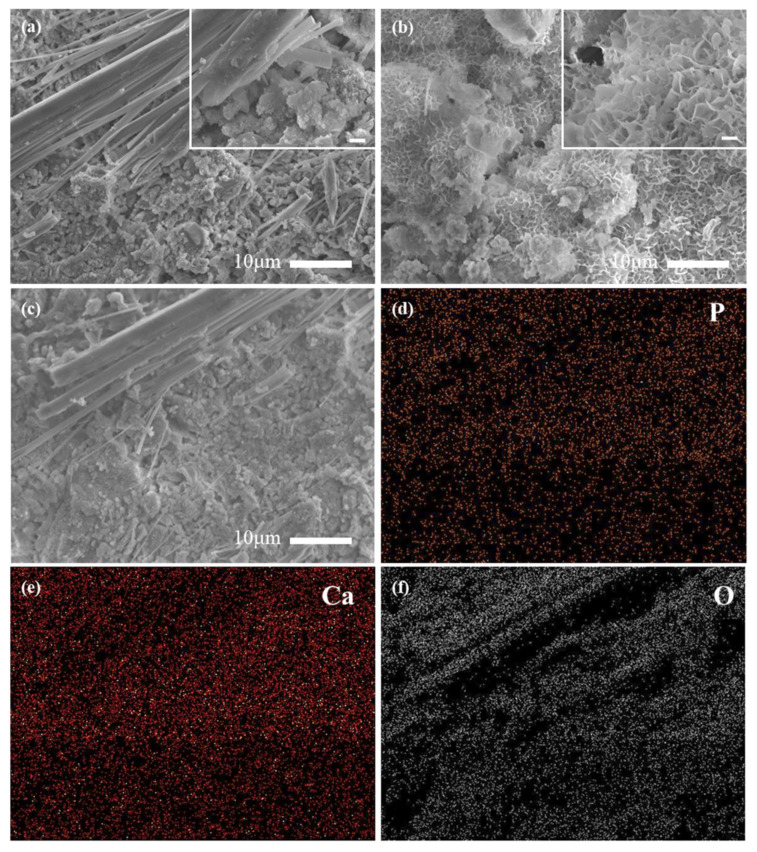
SEM morphologies and EDS mapping of SSD_P (10, 900): (**a**–**c**) show the morphologies at different sites with top right photos from a closer look at the said areas, respectively; (**d**–**f**) EDS mappings for P, Ca, and O elements, (marked in orange, red, and black) are demonstrated.

**Figure 4:**
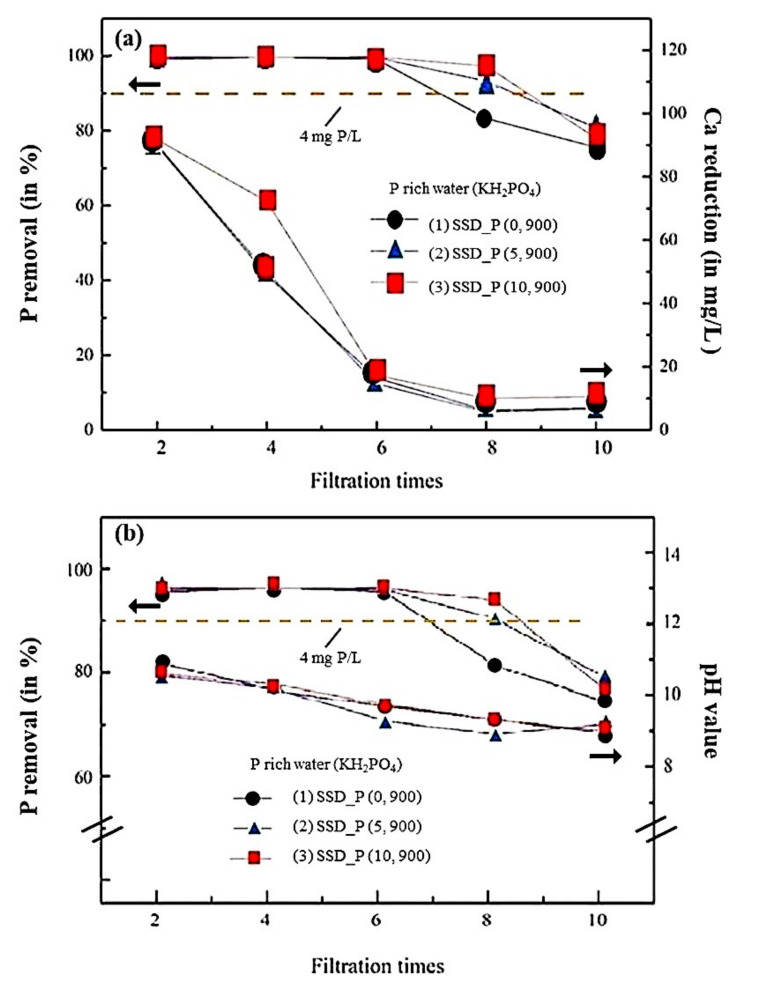
(**a**) The relations between filtering performances (P removal rates) and the released concentrations of Ca^2+^ as a function of filtering times are demonstrated for SSD_P (x, y) (x = 0, 5, 10 wt%, y = 900 °C). (**b**) The relations between filtering performances (P removal rate) and pH values in the filtrated water as a function of filtering times are demonstrated for SSD_P (x, y) (x = 0, 5, 10 wt%, y = 900 °C). The effluent standard limit of P ion under 4 mg/L is marked as an orange dash line.

The ladle steel slag powders with a particle size of ~100 μm (YIEH united steel corp., Kaohsiung, Taiwan) were used. Mixing ladle steel slag powder with 0, 5, 10 wt% of polyvinyl alcohol (PVA) powder. A porous steel slag disc could be formed by the P/M method. As illustrated in Figure 1a—(1)~(2), for one disc, ladle steel slag powder mixing with PVA powder of 0.5 g was compacted into a cylindrical disc of ~10 mm in diameter and ~5 mm in thickness; the compression stress was 200 MPa. The as-compacted sample is denoted as SSD_P (x’) (x’ = 0, 5, 10 wt% of PVA). The samples were heat-treated at 800, 900, or 1000 °C for 3 h, as shown in Figure 1a (3). The final samples are denoted as SSD_P (x, y) (x = 0, 5, or 10 wt%; y = 800, 900 or 1000 °C), as shown in Figure 1a—(4). The P-rich water filtration test is schemed in Figure 1b, while (5) is the simulated wastewater (P-rich water) with a concentration of 40 mg P/L, (6) is filtration procedure through the samples SSD_P (x, y) and soaking samples in P-rich water for 3 h, (7) is the collection of filtrated water, and (8) is the assessments of the tested SSD_P (x, y) and the filtrated water. The filtered water is sampled every 6 h within two testing cycles.

### 2.2. Quality Assessment of SSD_P (x, y) 

In Figure 2a–d, surface morphology of SSD_P (x, y) were sputtered with a layer of Pt and then characterized by a field-emission scanning electron microscope (FE-SEM, JSM-7000, JEOL, Tokyo, Japan) with an accelerating voltage of 10 kV, under a chamber vacuum of 5.15 × 10^−3^ Pa. The crystalline structure of SSD_P (x, y) was determined using X-ray diffraction with CuKα radiation (D2 Phaser, Bruker AXS Gmbh, Karlsruhe, Germany), as shown in Figure 2e. Figure 2f illustrates a compression stress test (AG-IS 100 kN, Shimadzu, Kyoto, Japan) for SSD_P (x, y). All tests have been run-up to a strain rate of 50% and relative compression stress was subsequently determined. The yield strength and relative strength (defined as the ratio of the strength of the porous material to that of the solid material) were then obtained. The resulting porosity measurement of disc samples was carried out using the method following Archimedes’ principle due to its experimental simplicity and reasonable reliability [36]. The mineral composition of SSD_P (x, y) was obtained by X-Ray Fluorescence (XEPOS, Spectro, XRF, Kleve, Germany) [37].

### 2.3. Assessment of Filtering Efficiency

The elemental composition mapping was determined using energy dispersive spectroscope (SEM-EDS; 7000, JEOL, Tokyo, Japan). As the energies of X-ray are the characteristic of the differences between the two shells and of the atomic structure of the emitting element, EDS allows the elemental composition of SSD_P (x, y) to be measured. To characterize the chemical compositions before/after the filtration, P removal (in %) and Ca reduction (in mg/L), in SSD_P (x, y) and P-rich water, inductively coupled plasma mass spectrometry (ICP-OES; ULTIMA, Jobin-Yvon, Horiba, Paris, France) and pH meter (PHM210, Radiometer Analytical SAS, Lyon, France) were employed [39]. All of the data presented is the average of measurements taken from six samples. 

## 3. Results and Discussion 

### 3.1. Physical Properties of SSD_P (x, y) 

Surface morphologies of SSD_P (x, y) are respectively shown in Figure 2a–d. With the increases of annealing temperature, recrystallization (e.g., marked in squares) and then grain growth (e.g., marked in open circles) of SSD_P (x, y) were observed. The sample SSD_P (10, 0) shows fragmentary, and SSD_P (10, 800) and SSD_P (10, 900) that had been undergone heat treatment are denser. The SD_P (10, 1000) shows the morphology and densification trend during the grain growth process, which is not conducive to the formation of connected holes. The XRD patterns of SSD_P (x, y) were respectively shown in Figure 2e. The results showed that Ca_2_SiO_4_ and CaSiO_3_ structures were found on all samples, while SSD_P (10, 800) and SSD_P (10, 900) were partially recrystallized. In addition, SSD_P (10, 1000) showed mostly recrystallized, so its mineral composition might have changed. Therefore, the XRD pattern and surface morphology of SSD_P (10, 1000) were different from those of SSD_P (10, 800) and SSD_P (10, 900).

In Figure 2f, SSD_P (x, y) with the added PVA powder in wt% (i.e., corresponding to the as-formed porosities) were associated with the measured compression stress. All the values were respectively averaged (N = 6). The compressive stress of SSD_P (0, 900), SSD_P (5, 900), and SSD_P (10, 900) resulted in a sharp decline (i.e., 23.4, 12.0 to 6.8 MPa) with the increase of porosity (i.e., 0, 5 to 10%). The maximum load stress of SSD_P (0, 900) is about four times larger than that of SSD_P (5, 900) or SSD_P (10, 900); the percentage of space holder thus affects the strength of compacted steel slags. According to ASTM C159-06 [38], the compression strengths of all three samples are generally suitable for use. Table 1 lists the performance of SSD_P (0, 900), SSD_P (5, 900), and SSD_P (10, 900) measured by Archimedes principle, including volume, bulk density, porosity. The results show that it is presumed that the porosity obtained by adding NaCl as a space-retaining agent has roughly uniformity. The thermal decomposition behavior of PVA, as shown in Supplementary Material, Figure S1. PVA vaporization temperature range is from 250 °C to 325 °C, proved that PVA can be removed during the first-stage heat treatment.

### 3.2. Chemical Composition of SSD_P (x, y)

The concentrations (in %) of the elements and main oxides obtained by XRF analysis are shown in Table 2. No harmful elements such as chromium (Cr) and sulfur (S) were detected in P-rich water. These test results indicate that under alkaline conditions (pH 11~9), the metals that are present in slag do not leach to any appreciable degree, and they should not be considered hazardous waste. Surface morphologies of the tested SSD_P (10, 900) were respectively shown in Figure 3a–c, followed by EDS mappings on P, Ca, and O elements for Figure 3d–f, respectively shown with the colors in orange, red, and black. The results showed that after filtration, the surface was partially covered by fibrous crystals and flocculated sediments, which was probably related to the zeolite and calcium-phosphorus hydrated mixture. The process of P removal may have two main mechanisms: (a) Formation of calcium phosphate phase in SSD_P (10, 900), mainly brushite; (b) Surface complexation with KOH and NH_4_OH groups of zeolite structure or unreacted minerals in SSD_P (10, 900) [40].

### 3.3. Filtering Performances of SSD_P (x, y) in the P-Rich Water (KH_2_PO_4_) 

In Figure 4a, the diagrams of filtering performances (i.e., shown as P removal (in %)), and Ca^2+^ (in mg/L) as a function of filtering times were demonstrated. The samples SSD_P (x, 900) with the porosities x = 0, 5, and 10 wt% in the P-rich water (KH_2_PO_4_) were compared. For the concentration of Ca^2+^ (CaO from SSD) in P-rich water, it decreases sharply with the increase of filtration time, and reaches the minimum value after six times of filtration in the filtered water, i.e., Ca^2+^ comes from the hydrolysis of CaO in SSD that may mostly be consumed after six filtration time. SSD_P (10, 900) with higher porosity showed relatively high released amount of Ca^2+^ in the filtrated water for four filtration times. After six times of filtration of these three samples, the Ca^2+^ concentration in the filtered water was low, which is probably due to the depletion of the releasable CaO in the SSD. 

In Figure 4a, the effluent standards limit of P under 4 mg/L (i.e., ~90% as shown in the figure) was drawn on the top as a dash line. For all three samples, the P removal rate remained at ~100% during six filtering times, which may represent the threshold for P removal. Thereafter, the filtration efficiency decreases as the filtration time increases. For SSD_P (0, 900) after eight times of filtering and SSD_P (5, 900) and SSD_P (10, 900) after 10 times of filtering, they failed to pass the standard. The results are in good agreement with the consumption of Ca^2+^, which is used for the precipitation of PO_4_^3−^ in the P-rich water. Due to the increase in porosity, the filtration efficiency of SSD_P (10, 900) is higher than that of SSD_P (0, 900) and SSD_P (5, 900), which can promote the hydrolysis of CaO to Ca^2+^ and react with PO_4_^3-^ in the P-rich water.

In Figure 4b, pH values, as compared with P removal of the filtrated water from Figure 4a, were measured. In general, P removal in the P-rich water under the alkaline environment tends to benefit the reaction of 3Ca^2+^ + 2PO_4_^3−^ = Ca_3_(PO_4_)_2_ [41]. The results showed that within 4 filtration times, because P removal was completed and the released Ca^2+^ remained in the filtrated water, the pH values exhibited higher than 10. After six filtration times, the released Ca^2+^ largely decreased and PO_4_^3−^ remained in the filtrated water, therefore the pH values of the filtrated water decreased to 8 (less alkaline), which may also be detrimental to the precipitation reaction.

In summary, by measuring the released concentrations of Ca^2+^, P removal rates, and pH values in the filtrated water of different filtration times, the filtering performance of SSD_P (10, 900) in the P-rich water (KH_2_PO_4_) is superior to that of SSD_P (0, 900) and SSD_P (5, 900) and the filtration times can reach to eight.

#### Filtering Performances of SSD_P (x, y) in the P-Rich Water (NH_4_H_2_PO_4_) 

**Figure 5:**
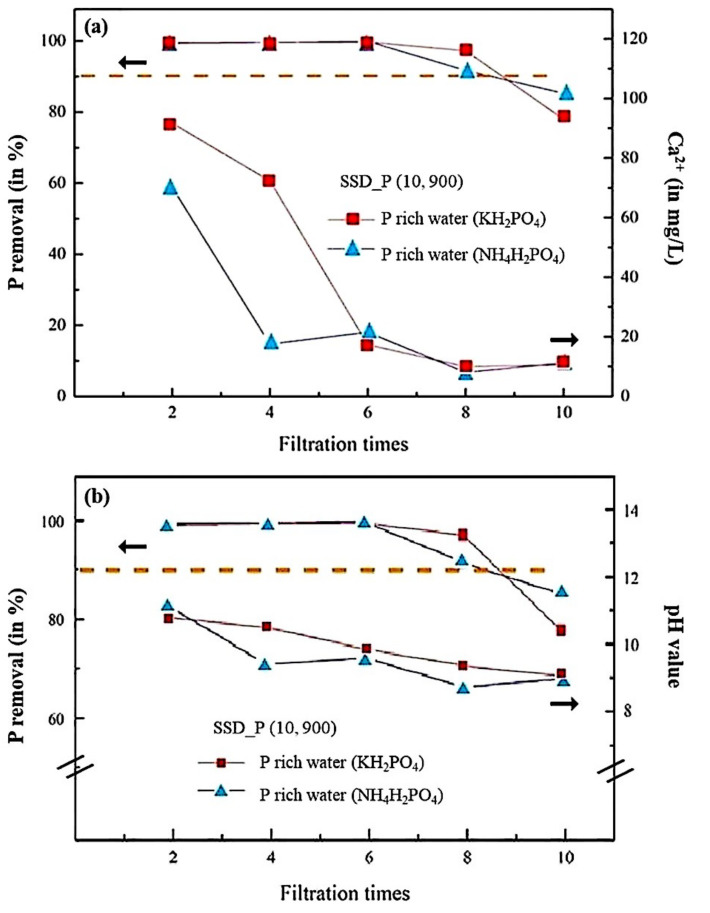
(**a**) The relations between filtering performances (P removal rates) and the released concentrations of Ca^2+^ as a function of filtering times are demonstrated for SSD_P (10, 900) in different P-rich water, (KH_2_PO_4_ or NH_4_H_2_PO_4_). (**b**) The relations between filtering performances (P removal rate) and pH values in the filtrated water as a function of filtering times are demonstrated for SSD_P (10, 900) in different P-rich water, (KH_2_PO_4_ or NH_4_H_2_PO_4_).

In Figure 5a,b, SSD_P (10, 900) was used for filtrating the P-rich water (NH_4_H_2_PO_4_) and compared its performance with the previous one. The release of Ca^2+^ in SSD_P (10, 900) exhibited different in the alkaline environment of NH_4_H_2_PO_4_. It has been reported that the reaction 3Ca^2+^ + 2PO_4_^3−^ = Ca_3_(PO_4_)_2_ is affected by the dissolution of NH_4_^+^ [42]. In Figure 5a, the concentration of Ca^2+^ in the filtrated water (NH_4_H_2_PO_4_) was significantly lower than that in the filtrated water (KH_2_PO_4_) for the filtration times of two and four. It is likely that either the release of Ca^2+^ is suppressed by the presence of NH_4_^+^ (case 1) or CaO in SSD reacts with NH_4_OH in the P-rich water (case 2) that reduces the concentration of Ca^2+^ in the filtrated water. On the other hand, P removal in the P-rich water (NH_4_H_2_PO_4_) showed the similar efficacy with that in the P-rich water (KH_2_PO_4_), but declined after 8 filtration times. 

In Figure 5b, for the P-rich water (NH_4_H_2_PO_4_), the P removal was completed within six filtration times, whereas the released Ca^2+^ greatly reduced in the filtrated water within four filtration times (shown in Figure 5a), the pH value also decreased. Therefore, it is likely that Ca^2+^ or NH_4_^+^ has been consumed, resulting in a lower alkaline environment, which is consistent with cases 1 and 2 derived from Figure 4a.

In summary, by measuring the released concentrations of Ca^2+^, P removal rates, and pH values in the filtrated water of different filtration times in SSD_P (10, 900), the filtering performance of SSD_P (10, 900) in the P-rich water (KH_2_PO_4_) is superior to that in the P-rich water (NH_4_H_2_PO_4_). It is most probably because of the participation of NH_4_^+^ or NH_4_OH in the P-rich water that may interfere the reactions of the release of Ca^2+^ (CaO) in SSD and the precipitation of Ca_3_(PO_4_)_2_. Nevertheless, further study on the reaction kinetics of SSD_P (x, y) for P-rich water filtration in an alkaline environment is still needed.

### 3.4. Proposed Reaction Mechanism and Perspective

**Figure 6:**
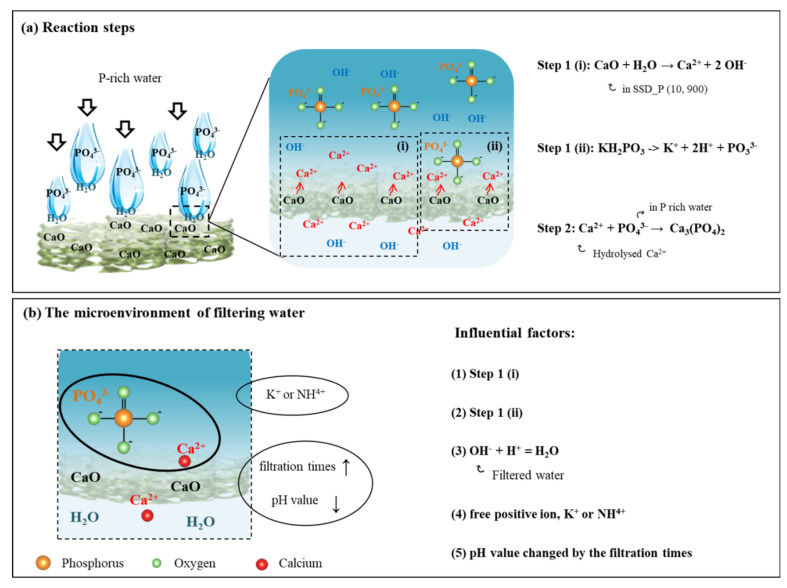
(**a**) The reaction mechanism for the release of Ca^2+^ and PO_4_^3-^ (step 1), and the precipitation of Ca_3_(PO_4_)_2_ (step 2) is illustrated. (**b**) The influential factors for the microenvironment of water that alters the filtration performances are suggested.

Based on the results of the filtering performances on SSD_P (x, y) in P-rich water, a reaction mechanism for P removal is proposed and illustrated in Figure 6. In Figure 6a, CaO in SSD is first hydrolyzed from the matrix of SSD (as shown in step 1 (i)), where high porosity of SSD and P-rich water (KH_2_PO_4_) are easy to release CaO to Ca^2+^ and simultaneously the hydrolysis of KH_2_PO_4_) (as shown in step 1 (ii)). The release of Ca^2+^ will react with PO_4_^3−^, and form the precipitate—Ca_3_(PO_4_)_2_ (as shown in step 2) [43]. In Figure 6b, five factors in the microenvironment of water are influential to P removal: In addition to the reaction steps 1 (i) (factor (1)) and (ii) (factor (2), there are three other factors: (3) the recombination of H^+^ and OH^-^ into H_2_O, (4) the kind of the simulated P-rich water with different free positive ion such as K+ or NH_4_^+^ that may also interfere the reactions of the release of Ca^2+^, and (5) pH value of the filtrating P-rich water decreased with the addition of filtration times. Note that as increased with the filtration times, the released amount of Ca^2+^ from CaO is reduced, followed by the coverage of the precipitated Ca_3_(PO_4_)_2_ on the surface or in the matrix of SSD. 

In particular, water plays an intermediate role in promoting the release of CaO from the matrix of SSD to Ca^2+^ and the dissociation of PO_4_^3−^ from KH_2_PO_4_ or NH_4_H_2_PO_4_. Due to changes in acidity and alkalinity of water by the filtration times and the presence of different free positive ion, K^+^ or NH^3+^, in P-rich water, the microenvironment of water accordingly alters the filtration performance for P removal. 

## 4. Conclusions

In this study, SSD_P (10, 900) in P-rich water (KH_2_PO_4_) provides relative high filtration performance and the filtration times can reach to eight. It is thus promising for reducing steel slags deposits as a porous compacted disc for P removal in P-rich water. The filtrated water meets the requirement of the effluent standard ISO 14001 [44], and the collected product—Ca_3_(PO_4_)_2_ in SSD is expected to be used for recycling, thereby realizing the application of circular economy, e.g., a part of Ca_3_(PO_4_)_2_ in SSD can be used as soil amendment or lime fertilizer. It is promising to reduce resource dependency, to increase the use of industrial by-products in protecting the environment, and to conserve natural resources.

## Figures and Tables

**Table 1:** The volumes, bulk densities, and porosities for SSD_P (x, y) (x = 0, 5, 10 wt%, y = 900 °C) are measured by Archimedes principle with 6 sampling numbers (n = 6).

PVA	Volume (cm^2^)		Bulk Density (g/cm^2^)		Porosity (%)	
(wt%)	Average	Deviation	Average	Deviation	Average	Deviation
0	0.6828	0.0055	1.4526	0.0112	28.9474	0.4520
5	0.6477	0.0034	1.4692	0.0151	36.1800	0.2975
10	0.6150	0.0040	1.4659	0.0043	42.8706	0.3903

**Table 2:** XRF data of element and oxide modes in % for different samples are measured.

	Element Mode (%)					Oxide Mode (%)			
	Sample 1	Sample 2	Sample 3	Sample 4		Sample 1	Sample 2	Sample 3	Sample 4
Ca	64.68	**✓**	**✓**	**✓**	CaO	74.54	**✓**	**✓**	**✓**
Si	32.95	**✓**	N.D.	N.D.	SiO_2_	21.37	**✓**	N.D.	N.D.
Mg	1	N.D.	N.D.	N.D.	MgO	0.9	N.D.	N.D.	N.D.
S	0.99	N.D.	N.D.	N.D.	SO_3_	1.5	N.D.	N.D.	N.D.
Cr	0.18	N.D.	N.D.	N.D.	Cr_2_O_3_	0.37	N.D.	N.D.	N.D.
Ti	0.1	N.D.	N.D.	N.D.	TiO_2_	0.27	N.D.	N.D.	N.D.
Fe	0.1	N.D.	N.D.	N.D.	Fe_2_O_3_	0.39	N.D.	N.D.	N.D.
P	-	N.D.	**✓**	**✓**	P_2_O_5_	-	N.D.	**✓**	**✓**

Sample 1—SSD_P (10, 900); Sample 2—after 8 filtration times of SSD_P (10, 900); Sample 3—at the beginning of 8 filtration times of P-rich water (KH_2_PO_4_); Sample 4—after 8 filtration times of P-rich water (KH_2_PO_4_); N.D.—under background noise.

## Data Availability

Not applicable.

## Supplementary Materials

The following are available online at https://www.mdpi.com/article/10.3390/ma14123187/s1, Figure S1: Thermal gravimetric analysis (TGA) SSD_P (x, 900), x = 0, 5, 10. Samples were subjected to heat treatment to test the PVA vaporization temperature range.
