# Supplementary material for: Recycled Steel Slag as a Porous Adsorbent to Filter Phosphorus-Rich Water with 8 Filtration Circles
Source: Materials (Basel). 2021 Jun 9;14(12):3187. doi: 10.3390/ma14123187 (PMC8228387; doi:10.3390/ma14123187)
Supplement: Supplementary file 1 [file materials-14-03187-s001.zip › materials-1246299-supplementary.pdf]

materials

Supplementary Materials

Recycled Steel Slag as a Porous Adsorbent to Filter Phosphorus-Rich Water With 8 Filtration Circles

Han Lee [1], Yen-Ling Peng [1], Liang-Ming Whang [2] and Jiunn-Der Liao [1,*]

The thermal decomposition behavior of PVA, as shown in Supporting data 1. PVA vaporization temperature range is from 250 °C to 325 °C, proved that PVA can be removed during the first-stage heat treatment.

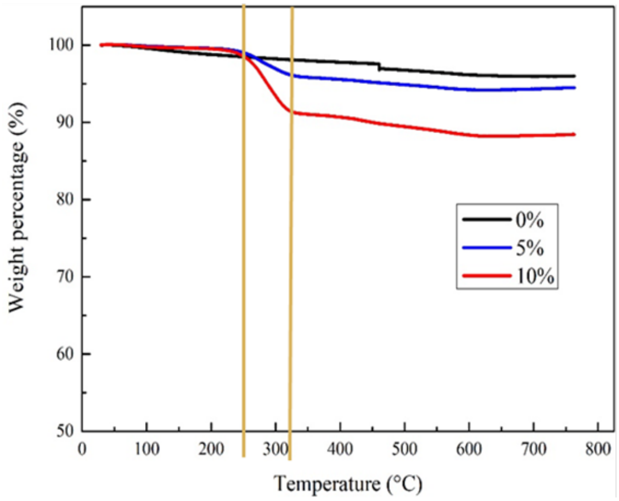

Figure S1. 1. thermal gravimetric analysis (TGA) SSD_P (x, 900), x = 0, 5, 10. Samples were subjected to heat treatment to test the PVA vaporization temperature range.
